# Do White Noise or Music Relieve Pain Caused by Botulinum Toxin Injections?

**DOI:** 10.1155/prm/3007685

**Published:** 2026-05-07

**Authors:** Zuleyha Ozgen

**Affiliations:** ^1^ Department of Dermatology, Acibadem Hospital, Atasehir, Istanbul, Turkey, acibadem.com.tr

**Keywords:** botulinum toxin, music, pain, white noise

## Abstract

**Objective:**

To investigate the impact of music and white noise on the pain caused by cosmetic botulinum toxin injections.

**Patient and Method:**

Seventy‐six participants between the ages of 18 and 45 who requested cosmetic botulinum toxin application to the upper face were enrolled in the study in three groups: favorite music (20), white noise (31), and control (25). The first and second groups listened to their favorite music and white noise, respectively, during and 10 min after the procedures, while the control group was exposed to background noise only. The participants rated their pain levels during the procedure and the impact of the sound on their stress level using a visual analog scale ranging from 0 to 10. Additionally, the participants in both the music and white noise groups were surveyed regarding their preferences for sound intervention for future botulinum toxin procedures.

**Result:**

The pain scores (95% confidence interval) for the control, music, and white noise groups were 6.80 [6.37–7.23], 5.7 [5.13–6.27], and 5.52 [4.99–6.04], respectively. Both the white noise group and the music group had significantly lower pain scores compared to the control group. Furthermore, individuals receiving botulinum toxin for the first time reported significantly higher pain scores than those who had prior experience with the treatment.

**Conclusion:**

Listening to white noise or music during cosmetic botulinum toxin injections can effectively reduce procedure‐related pain. However, further studies are required to reveal the mechanism of action of sound applications in pain management during cosmetic procedures and to determine the selection of appropriate candidates and specific application conditions.

## 1. Introduction

Botulinum toxin (Btx) is an exotoxin produced by the Gram‐positive anaerobic bacterium *Clostridium botulinum*. It binds to cholinergic receptors at the neuromuscular junction and inhibits the release of acetylcholine, causing flaccid paralysis. In dermatology, it is used to improve the appearance of dynamic wrinkles caused by facial expressions and to treat hyperhidrosis [1]. Patients often experience anxiety related to the use of needles during medical procedures, which may cause them to postpone treatment. Therefore, minimizing pain and discomfort during injections is an important consideration for healthcare providers [2].

It has been shown that music, which is a noninvasive, easily applicable, and safe application, can be effective in procedural, acute, or chronic pain [3]. A 2016 meta‐analysis of 97 studies found that music resulted in a statistically significant decrease of 1.13 units in pain visual analog scale (VAS) scores (0–10), reduced emotional stress caused by pain, improved physiological measures such as heart rate and blood pressure, and reduced the need for analgesics [3]. As far as we could find, only one study examined the effect of music on the pain of a Btx procedure and found that patients with chronic migraine who listened to classical music during onabotulinum toxin injections experienced less pain [4]. Although the mechanism by which music affects pain has not yet been clarified, it has three main effects: cognitive, emotional, and neurobiological. Functional MRI studies demonstrated that listening to music can cause activation differences in the anatomical regions involved in pain modulation when participants are given painful stimuli. Music provides a cognitive effect by distracting the patient’s attention from the procedure. Furthermore, some studies have shown that emotional factors, such as pleasure and joy, can effectively impact pain [5]. In a comparison between relaxing music, favorite music, and white noise, participants reported reduced pain sensitivity and catastrophic thinking about pain when listening to their favorite music, as opposed to relaxing music or white noise [6]. This suggests that the emotional connection between a person and their preferred music may be a contributing factor to its effectiveness in pain control [6, 7].

White noise is a sound that combines all frequencies with equal and constant intensity within the range of human hearing (20–20,000 Hz). Examples of white noise include the sound of a hair dryer, air conditioner, vacuum cleaner, fan, sea waves, and rain [8]. White noise has shown potential benefits for tinnitus patients by masking sounds, improving cognitive function, and calming neuropsychiatric patients [8, 9]. Recent studies suggest that white noise may also be effective for pain relief. A study of healthy newborns revealed that the group exposed to white noise during heel prick blood collection had significantly lower procedural behavioral pain scores and crying times [10].

Various analgesic modalities, including topical anesthetics and cold application, have been used to make minor interventional procedures, such as Btx injections, less painful and more comfortable, with varying degrees of success [1, 11]. While studies have shown the analgesic effects of white noise and music, there are insufficient data to support their analgesic effect during cosmetic Btx applications. The objective of this study is to examine the efficacy of white noise and music in alleviating pain and anxiety caused by facial Btx procedures.

## 2. Method

### 2.1. Patients

This prospective and controlled study was conducted in a private plastic surgery clinic. The study included patients aged 18–45 who received Btx treatment for cosmetic purposes to address wrinkles on the forehead, around the eyes, and the glabella. The patients were divided into three groups: favorite music, white noise, and control. A power analysis was performed to estimate the number of subjects required for the study. It was hypothesized that the mean pain score of the music or white noise groups would be 10% lower than that of the control group, as there are no previous studies evaluating Btx pain. With a 95% confidence interval (CI) and 80% power, the number of subjects in each group was calculated to be at least 16, with a planned enrollment of 25 subjects per group after consideration of dropouts.

Patients undergoing regular treatments at the clinic were informed about the study and were invited to participate. After obtaining written informed consent from all participants, they were included consecutively in the following three groups, in order of participation in the study: the control group, the music group, and the white noise group.

### 2.2. Procedures

The procedures were performed by a plastic and reconstructive surgery specialist with extensive experience and certification from the European Board. The same physician injected all subjects individually in separate time periods with the same trademarked toxin using a 32‐gauge needle with the assistance of the same nurse and under identical examination room conditions. The Dysport vials containing 500 U of abobotulinum toxin A were reconstituted with 2.5 cc of normal saline. The clinician performing the procedure determined the dosage based on the patient’s cosmetic needs. All patients received a topical cold application with an ice pack before and after the procedure.

Patients in the music group were allowed to listen to their preferred music or songs, beginning 10 min before and continuing throughout the procedure, while the white noise group listened to white noise during the same period as the music group. The control group was exposed only to background sound. The auditory stimuli, namely music and white noise, were played openly in the treatment room. The white noise stimuli for each participant were accessed from the same YouTube channel as the hair dryer sound (https://youtu.be/eJT8xuI_5PY?si=fFiXLAhoq-dQkHDn). The auditory stimuli were played at an intensity of 60 dB, at a distance of 150 cm from the patient’s chair. The audio levels were modified using a specific mobile application.

The VAS was explained verbally to the participants, who then were requested a rating of the pain experienced during the Btx injection on a VAS ranging from 0 to 10 (0 indicates no pain, and 10 indicates the most severe pain). Furthermore, participants in the music and white noise groups were asked to rate the effectiveness of the sound in reducing their stress levels on a VAS of 0–10 (0 indicating no reduction, 10 indicating complete elimination) and their preference for this sound intervention in future procedures, with response options of “yes,” “no,” or “not important.” Subjects with contraindications to Btx injection, such as myasthenia gravis, pregnancy, lactation, and those who had undergone any additional procedures in the area where Btx was injected, were excluded from the study.

### 2.3. Statistical Analyses

Statistical analyses were performed using IBM SPSS Statistics Version 26.0 (IBM Corp., Armonk, NY, USA). Demographic characteristics including age and sex were initially examined using descriptive statistics. Categorical variables were analyzed using the chi‐square test or Fisher’s exact test, as appropriate. The Kolmogorov–Smirnov test was applied to assess the normality of distribution for continuous variables. As the data did not follow a normal distribution, nonparametric tests were employed for subsequent analyses. The Kruskal–Wallis test was used to compare the three groups (control, music, and white noise). When statistically significant differences were detected, post hoc pairwise comparisons were performed using the Mann–Whitney *U* test with Bonferroni correction to adjust for multiple comparisons. For these post hoc Mann–Whitney *U* tests, effect sizes were calculated using the effect size coefficient *r* (*r* = *Z*/√N), and values of approximately 0.10, 0.30, and 0.50 were interpreted as small, medium, and large effects, respectively. All statistical tests were two‐tailed, and a *p*‐value of less than 0.05 was considered statistically significant.

### 2.4. Ethics

The study was approved by the Istanbul Prof. Dr. Cemil Tascioglu City Hospital Clinical Research Ethics Committee (Approval No.: E‐48670771–514.99‐232118778).

## 3. Results

The study’s participants comprised 83 individuals, including seven males and 76 females. However, given the limited number of male participants, the decision was made to exclude them from the study in order to enhance the homogeneity of the sample and ensure the validity of the statistical analyses. The demographic, clinical, and treatment‐related characteristics of the study groups are detailed in Table 1. The average ages of the participants were 41.2 (±7.14), 39 (±7.82), and 37.71 (±4.85) years for each respective group. Additionally, 36% (9), 35% (7), and 22.6% (7) of participants in each respective group reported previous experience with Btx. The groups did not differ significantly in terms of previous experience with Btx.

**TABLE 1:** Demographic and clinical characteristics of the study groups.

		**Control *N*:25**	**Music *N*:20**	**White noise *N*:31**	**p**

Age (mean [±SD])		41.2 (±7.14)	39 (±7.82)	37.71 (±4.85)	0.113

Additional disease (%)	Yes	1 (4)	2 (10)	2 (6.5)	0.722
	No	24 (96)	18 (90)	29 (93.5)	

Regularly used medication and supplement (%)	Yes	6 (24)	4 (20)	5 (16.1)	0.762
	No	19 (76)	16 (80)	26 (83.9)	

Botulinum toxin dosage (mean [95% CI]) (U)		126.4 [118.9–133.8]	109 [103.8–114.2]	106.77 [103.7–109.8]	0.000^∗^

Previous botulinum toxin experience (%)	Yes	16 (64)	13 (65)	24 (77.4)	0.480
	No	9 (36)	7 (35)	7 (22.6)	

Pain score (VAS) (mean [95% CI])		6.80 [6.37–7.23]	5.7 [5.13–6.27]	5.52 [4.99–6.04]	0.001^∗^

Pain score (VAS) of Btx‐experienced participants (mean [95% CI])		6.44 [5.89–6.99]	5.46 [4.7–6.23]	5.42 [4.78–6.05]	0.048^∗^

Pain score (VAS) of Btx‐naïve participants (mean [95% CI])		7.44 [6.89–8]	6.14 [5.15–7.13]	5.86 [4.73–6.98]	0.01^∗^

Effects of sound on stress level (VAS) (mean [95% CI])			6.35 [5.2–7.5]	7.19 [6.51–7.87]	0.314

Preference for subsequent procedures (%)	Yes	—	17 (85)	30 (96.8)	0.161^a^
	No	—	0	1 (3.2)	
	Not important	—	3 (15)	0	

*Note:* Btx: botulinum toxin.

Abbreviations: CI, confidence interval; SD, standard deviation; VAS, visual analog scale.

^a^When combining the “no” and “not important” groups as “non‐yes” and comparing them with the “yes” group.

^∗^ *p* < 0.05 indicates statistical significance.

The pain scores (95% CI) for the control, music, and white noise groups were 6.80 [6.37–7.23], 5.7 [5.13–6.27] and 5.52 [4.99–6.04], respectively. A statistically significant difference was observed in VAS pain scores across the three groups (*p* = 0.001, df = 2). Subsequent post hoc analyses indicated that both the white noise and music groups exhibited significantly lower pain scores in comparison to the control group (music *p* = 0.003, *r* = −0.44; noise *p* = 0.001, *r* = −0.445).

The mean (95% CI) pain score of participants receiving their first injection was 6.57 [6.05–7.08], which was significantly higher than the score of participants with previous Btx experience (5.74 [5.36–6.11], *p* = 0.015). Upon analyzing the association between pain scores and Btx experience condition in each study arm separately, no significant association was found in the music and white noise arms (music *p* = 0.241; noise *p* = 0.473). However, in the control arm, there was a significant difference between the pain scores of first‐time injection participants and experienced participants (*p* = 0.023, *r* = −0.475).

Among the first‐time–exposed participants, a significant difference in pain scores was observed across the study groups (df = 2, *p* = 0.01). The mean pain score for the noise group and the white noise group was significantly lower than that of the control group (noise *p* = 0.012, *r* = −0.65; music *p* = 0.016, *r* = −0.626). However, there was no significant difference between the music and noise groups (*p* = 0.71). Among Btx‐experienced participants, a significant difference in pain scores was found only between the control and noise groups (*p* = 0.027, *r* = −0.355).

The dose (95% CI) of Btx was 126.4 [118.9–133.8] U in the control group and 109 [103.8–114.2] and 106.77 [103.7–109.8] U in the music and white noise groups, respectively. Although the dose was comparable between the music and white noise groups (*p* = 0.440), the mean dose in the control group was significantly higher than in the other groups (noise *p* = 0.001, music *p* = 0.001). However, no significant correlation was found between the dose and pain scale (Spearman’s correlation, *p* = 0.734). The partial correlation, which was controlled for the study group, was also not significant (*p* = 0.128).

The mean score (95% CI) for the positive effect of music on stress was 6.35 [5.2–7.5], while that of white noise on stress was 7.19 [6.51–7.87]. There was no statistically significant difference between the two (*p* = 0.314). Additionally, a significant negative correlation was found between the effect of sound intervention on stress and the pain scale (*p*: 0.000, Spearman’s *r*: 0.742) in the white noise group.

In the music group, 85% (17/20) of participants preferred the sound application for their next procedure compared to 96.8% (30/31) in the white noise group (*p* = 0.161). One participant in the white noise group reported that the sound disturbed her and expressed a preference for its absence in future procedures. The remainder expressed that the presence of sound was unimportant.

## 4. Discussion

This prospective observational study evaluated the effects of music and white noise on pain experienced during Btx injections in 76 participants. Ice packs were applied topically to all participants before and after the procedure. The groups that listened to their preferred music or white noise were compared with the control group that underwent the procedure under background sound only. Both the white noise and the music groups had significantly lower pain scores compared to the control group. Furthermore, the first‐time experience of the Btx injection significantly increased the pain felt. Most participants reported that the application of sound during the procedure had a positive effect on their stress levels. Additionally, the majority expressed a preference for sound to be used in future procedures.

People may feel anxiety due to pain during cosmetic Btx procedures, which could lead to postponement of the procedure. Various methods can be used to alleviate pain, such as using smaller needle tips and changing them frequently, using preserved saline as a diluent, applying topical anesthetic creams, using cryoanesthesia with ice packs or cooled air, employing vibration anesthesia, and skin pinching [1, 2, 11, 12].

Research into music‐induced analgesia has demonstrated that music can have an analgesic effect on acute, chronic, postoperative, or experimental pain [3, 5]. The study of Mitchell and MacDonald, using experimental cold pressor pain, examined the effects of listening to favorite music, relaxing music, and control white noise on tolerance, pain intensity scores, and perceived control. In the favorite music group, pain tolerance was found to be significantly longer compared to relaxation music and white noise, and perceived control was also found to be significantly higher. However, only in the female group, pain intensity scores were significantly lower in the favorite music and relaxation music arms compared with white noise [7]. In the present study, which included only female participants, pain scores were significantly lower in the music arm compared to the control arm, despite the proximity of their respective CIs. Pain‐related fear and catastrophizing are important factors in pain perception. Neuropsychological differences between men and women have been reported in the process of pain perception, with women exhibiting greater medical fear of pain and catastrophizing [13–15]. The pain‐relieving effect of favorite music works, at least in part, through emotional pathways such as catastrophic thinking. This may have led to different pain outcomes in men and women [6, 13, 15]. Although some experimental studies have reported that women are more sensitive to pain and report higher pain scores, it is important to note that this may be related to the experimental method and social context [7].

White noise has been shown to regulate emotions, enhance visual and auditory sensations, alleviate pain and anxiety, and induce sedation [9]. In a study of 60 newborns in the intensive care unit, Çetinkaya et al. found that playing white noise during and 30 s after invasive procedures can alleviate pain, as evidenced by behavioral pain scores, and reduce the duration of crying. This study demonstrated the potential benefits of using white noise in pain management for newborns [16]. Currently, there is no study on the effects of white noise on pain during cosmetic procedures. However, our study suggests that listening to white noise during cosmetic Btx procedures can significantly reduce pain scores.

Anxiety and fear of pain are the factors that can exacerbate the severity of pain. A study conducted on medical university students, using the Fear of Pain Questionnaire‐9, found that as the students gained more experience, their fear of pain decreased [13]. Pain experience has been reported to affect cognitive and emotional processes related to pain and, in some cases, reduce pain sensitivity [17, 18]. Furthermore, Yoshida et al. demonstrated that pain perception can be modulated by predictability, with uncertainty leading to an increase in perceived pain [19]. The current study found that participants receiving their first Btx injection reported significantly higher levels of pain than those with prior experience. In light of previous reports, this may be due to uncertainty in predicting pain and a potentially high fear of pain. Furthermore, since the hyperalgesic effect of the first experience on pain perception was not associated with pain scores in the music and noise groups but only in the control group, it can be indicated that white noise and music applications may modify this first‐experience effect.

Previous studies have shown that music and white noise can reduce anxiety scores [3, 20, 21]. In their study on 75 adult patients undergoing spinal anesthesia, Ilkkaya et al. reported significantly lower postoperative anxiety scores and significantly higher patient satisfaction in the white noise and music groups compared to the ambient noise group [21]. Although the anxiety rating scale was not used in our study, patients in both the music and noise groups reported a positive effect of sound interventions on their stress levels. Additionally, the majority of patients expressed a preference for the sound intervention in future procedures.

The study’s limitations include a restricted sample size, the unavailability of validated anxiety and pain catastrophizing scales, and the lack of a standardized protocol for measuring perceived sound levels.

Although translating statistical differences into clinical significance is challenging, partly due to potential biases such as the unconscious encouragement of patients receiving auditory stimulation, the observed between‐group differences suggest that auditory stimulation may play a meaningful role in pain management in routine clinical practice. The use of low‐cost, safe interventions—such as white noise or music—in combination with established modalities like cryoanalgesia may enhance patient comfort during cosmetic procedures. However, further research is necessary to uncover the mechanism of action of sound applications in pain management during minimally invasive cosmetic procedures and to determine the specific patient and application conditions required to achieve optimal effectiveness.

## Funding

No funding was received for this manuscript.

## Conflicts of Interest

The author declares no conflicts of interest.

## Data Availability

The data that support the findings of this study are available from the corresponding author upon reasonable request.
